# Exposure of an occluded hemagglutinin epitope drives selection of a class of cross-protective influenza antibodies

**DOI:** 10.1038/s41467-019-11821-6

**Published:** 2019-08-28

**Authors:** Yu Adachi, Keisuke Tonouchi, Arnone Nithichanon, Masayuki Kuraoka, Akiko Watanabe, Ryo Shinnakasu, Hideki Asanuma, Akira Ainai, Yusuke Ohmi, Takuya Yamamoto, Ken J. Ishii, Hideki Hasegawa, Haruko Takeyama, Ganjana Lertmemongkolchai, Tomohiro Kurosaki, Manabu Ato, Garnett Kelsoe, Yoshimasa Takahashi

**Affiliations:** 1 Department of Immunology, National Institute of Infectious Diseases, 1-23-1 Toyama, Shinjuku, Tokyo, 162-8640 Japan; 2 Department of Life Science and Medical Bioscience, Waseda University, 2-2 Wakamatsucho, Shinjuku, Tokyo, 162-8480 Japan; 3 Center for Research and Development of Medical Diagnostic Laboratories (CMDL), Faculty of Associated Medical Sciences, Khon Kaen University, 123 Mittraphap Road, Khon Kaen, 40002 Thailand; 4 Department of Immunology, Duke University, Durham, NC 27710 USA; 5 Laboratory of Lymphocyte Differentiation, WPI Immunology Frontier Research Center and Graduate School of Frontier Biosciences, Osaka University, 3-1 Yamadaoka, Suita, Osaka, 565-0871 Japan; 6 Influenza Virus Research Center, National Institute of Infectious Diseases, 4-7-1 Gakuen, Musashimurayama, Tokyo, 208-0011 Japan; 7 Department of Pathology, National Institute of Infectious Diseases, 1-23-1 Toyama, Shinjuku, Tokyo, 162-8640 Japan; 8 Department of Clinical Engineering, Chubu University College of Life and Health Sciences, 1200 Matsumoto, Kasugai, 487-8501 Aichi, Japan; 9 Laboratory of Immunosenescence, Center for Vaccine and Adjuvant Research, National Institutes of Biomedical Innovation, Health and Nutrition, 7-6-8 Saitoasagi, Ibaraki, Osaka, 567-0085 Japan; 10 Center for Vaccine and Adjuvant Research, National Institutes of Biomedical Innovation, Health and Nutrition, 7-6-8 Saitoasagi, Ibaraki, Osaka, 567-0085 Japan; 11 Division of Vaccine Science, Department of Microbiology and Immunology, The Institute of Medical Science, The University of Tokyo, 4-6-1 Shiroganedai, Minato, Tokyo, 108-8639 Japan; 12 Laboratory for Lymphocyte Differentiation, RIKEN Center for Integrative Medical Sciences, 1-7-22 Suehirocho, Tsurumi, Yokohama, Kanagawa 230-0045 Japan; 13 Department of Mycobacteriology, National Institute of Infectious Diseases, 4-2-1 Aobacho, Higashimurayama, Tokyo, 189-0002 Japan; 14 Human Vaccine Institute, Duke University, Durham, NC 27710 USA

**Keywords:** Antibodies, Vaccines, Viral host response, Influenza virus

## Abstract

Germinal center (GC) B cells at viral replication sites acquire specificity to poorly immunogenic but conserved influenza hemagglutinin (HA) epitopes. Here, high-throughput epitope mapping of local GC B cells is used to identify conserved HA epitope selecting cross-reactive antibodies that mediate heterosubtypic protection. A distinct feature of this epitope is an occlusion in the naive trimeric HA structure that is exposed in the post-fusion HA structure to occur under low pH conditions during viral replication. Importantly, systemic immunization by the post-fusion HA antigen results in GC B cells targeting the occluded epitope, and induces a class of protective antibodies that have cross-group specificity and afford protection independent of virus neutralization activity. Furthermore, this class of broadly protective antibodies develops at late time points and persists. Our results identify a class of cross-protective antibodies that are selected at the viral replication site, and provide insights into vaccine strategies using the occluded epitope.

## Abstract (web summary)

Antibody cross-reactivity can help to prevent escape mutations from enabling viral escape, but underlying mechanisms are unclear. Here the authors identify influenza hemagglutinin epitopes that are exposed during viral replication and which result in the generation of a class of protective cross-reactive antibodies.

## Introduction

Viruses have acquired several mechanisms to evade antibody surveillance as a result of the host/pathogen co-evolution, one of which is the acquisition of escape mutations. The counteraction of humoral responses against the escape mutations is to acquire sufficient breadth of antibody specificity to include escape variants and to preserve cross-reactive antibody production in long-lived memory compartments (memory B cells and long-lived plasma cells)^1^. Antibody breadth or cross-reactivity is achieved by two different pathways: germline-encoded cross-reactivity for variable viral epitopes or somatic evolution in germinal centers (GCs) for conserved viral epitopes^2^.

Previous observations of an early, GC-independent pathway for recruiting germline-encoded antibodies into the memory compartment led to a proposal that low-affinity/cross-reactive B cells act to increase the breadth of humoral protection^3–5^. This early, germline cross-reactivity is associated with B cell antigen receptor (BCR)-mediated recognition of variable epitopes with low affinity and supported by the intriguing findings of McGargill’s group showing that limiting the generation of highly specific IgG B cells by rapamycin treatment enhanced the breadth of protective antibody responses to influenza^6^.

Protective cross-reactivity is also acquired by targeting conserved viral epitopes that are often immunosubdominant and poorly immunogenic. This process requires the improvement of BCR affinity and specificity through V(D)J mutation and selection within GCs^7^. Extensive analyses of HIV broadly neutralizing antibodies (bNAbs) has highlighted the requirement for extraordinary frequencies of V(D)J mutations and repeated rounds of selection to acquire the requisite affinity/specificity for conserved, immunosubdominant neutralizing epitopes^8^. Likewise, influenza bNAbs that target the hemagglutinin (HA) conserved epitopes acquire the affinity/specificity through the accumulation of V(D)J mutations^9–11^, albeit to a lesser extent than HIV bNAbs. Thus, V(D)J mutations and selection during the GC pathway fine-tune the specificity and affinity of the antibodies to conserved influenza epitopes. However, GC selection for conserved epitopes appear to be less robust or competitive than that for immunodominant and variable epitopes. The mechanisms for this dichotomy remain elusive and constitute a key issue for developing cross-protective vaccines against rapidly mutating viruses.

By monitoring B cell responses targeting conserved HA epitopes in mice, we previously demonstrated that influenza virus infection induces ectopic formation of lung GCs at the viral replication site, where cross-reactive B cells are selected at increased frequencies^12^. This result prompted us to identify the conserved epitopes that are utilized as selecting antigens and the mechanism of their enhanced immunogenicity. Here, we apply a single cell culture method that allows high-throughput analysis of B cell epitopes in humans and mice^13,14^. We identify long alpha helix (LAH) epitopes in the HA2 stem regions, which are normally occluded in native HA form, as the selecting epitope for cross-reactive B cells in local GCs. Moreover, we demonstrate that post-fusion HA antigen exposes the LAH epitope to increase its antigenicity and serves as the selecting antigen for the LAH-binding B cells; the consequence of these events is robust and durable elicitation of cross-protective LAH IgG. Thus, GC B cells at viral replication site utilize antigenic plasticity for guiding the specificity to occluded epitopes that potentially include virus vulnerable sites.

## Results

### Single B cell cultures validate specificity of GC B cells

**Fig. 1:**
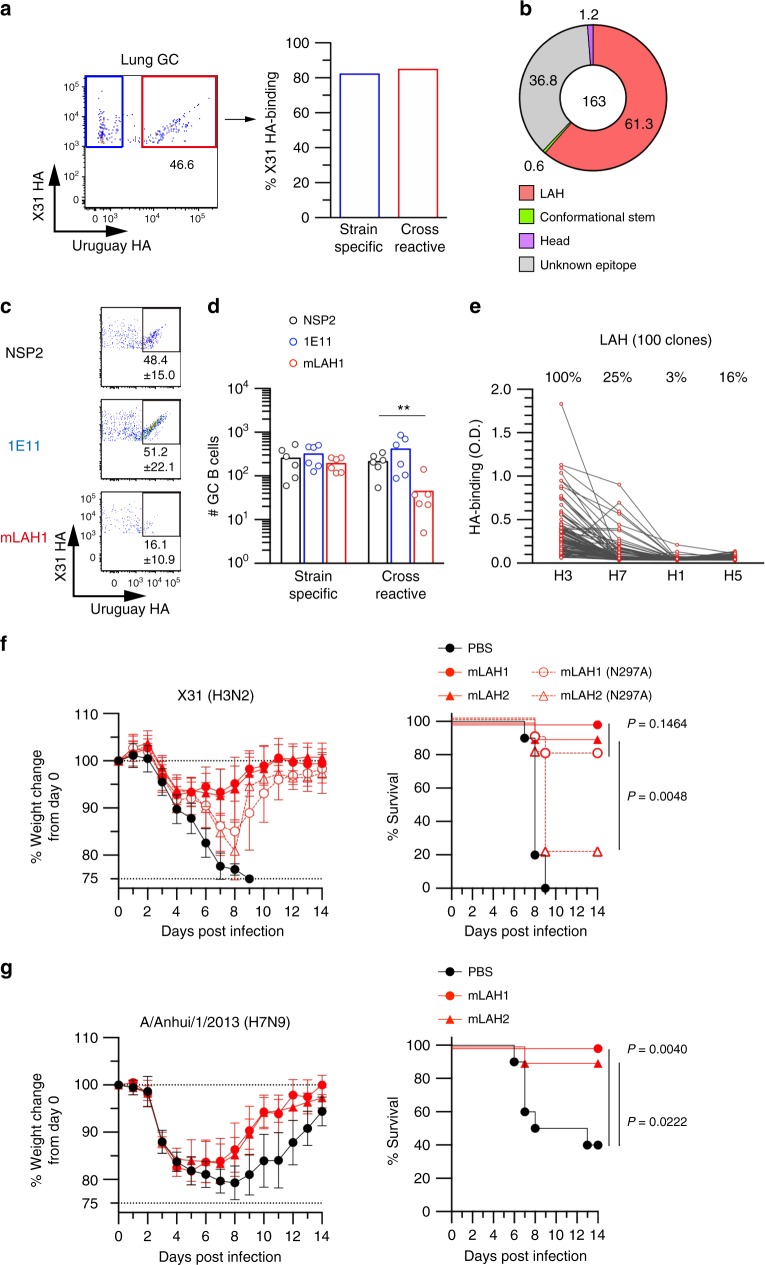
LAH is a selecting epitope for cross-reactive GC B cells at viral replication site. **a** X31 HA-binding GC B cells in the lungs were recovered from X31-infected BALB/c mice at day 20 after infection and separated into strain-specific (blue) and cross-reactive (red) cells. Antigen specificity of both populations were evaluated by using mAbs in culture supernatants of single cell cultures. **b** High-throughput epitope mapping analysis was applied to the cross-reactive GC B cells (X31/Urg HA binders) in the lungs. The data from pooled 36 mice is shown. **c** Cross-reactive GC B cells were enumerated in the lungs 5 days after the injection of LAH-binding IgG1 (mLAH1), head-binding IgG1 (1E11), and non-binding IgG1 (NSP2). **d** The number of strain-specific and cross-reactive GC B cells were determined by flow cytometry 5 days after antibody treatment. Each circle represents the result from an individual mouse. The combined data from two independent experiments are shown. **e** Heterosubtypic reactivity of H3 LAH-binding clones was evaluated by ELISA using rHA as coating antigens. H3; X31, H7; A/Anhui/1/2013, H1; A/Narita/1/2009, H5; NIBRG-14 (originally from A/Vietnam/1194/2004). **f** Protective function of LAH-binding IgG2a and N297A mutant (100 μg per mouse) was assessed after lethal challenge by X31 (5 × LD50). **g** Heterosubtypic protection by LAH-binding IgG2a (100 μg per mouse) was assessed after H7N9 challenge (A/Anhui/1/2013, 1 × LD50). The combined data from two independent experiments (*n* = 10 per group) are shown. Left; body weight loss. Right; survival curve. Values represent mean ± s.d. **P < 0.01. The *P*-values were determined with a two-tailed Mann–Whitney test **d** and log-rank test **f**, **g**. Source data are provided as a Source Data file

Using two rHA probes of the same H3 subtype (X31 and A/Uruguay/716/07), we detected strain-specific (X31 single positive) and cross-reactive (X31/Urg double positive) GC B cells from the lungs of X31-infected mice, as previously described (Fig. 1a)^12^. The specificity of our staining procedure was thoroughly validated using mouse B cells from naive mice and those from BCR-knock-in mice to an unrelated hapten antigen^12^. In addition, we confirmed the antigen specificity of identified GC B cells using single-cell Nojima cultures^13^.

In Nojima cultures, lung GC B cells had lower cloning efficiencies than did phenotypically similar GC B cells from secondary lymphoid tissues^13^ (Supplementary Table 1), perhaps the consequence of the harsher conditions necessary for the recovery of B cells from lung tissue. We analyzed HA-binding frequencies of the clonal IgGs secreted by strain-specific (69 clones) and cross-reactive (191 clones) GC B cells (Fig. 1a). The concordance of specificity by flow cytometry and clonal IgG binding was high; 83% (57/69) strain-specific GC clones secreted IgG reactive with only X31 HA and 85% (163/191) of cross-reactive clones exhibited the appropriate dual reactivity. We speculate that these specific HA-binding ratios underestimate the actual ratios, because low affinity, soluble antibody may be undetectable by ELISA, even though the membrane-bound form can be detected by flow cytometry using antigen probes^15^. Thus, our previous data and Nojima culture data in this study validate the specificity of our HA probes to identify strain-specific and cross-reactive GC B cells.

### LAH is a selecting epitope of cross-reactive GC B cells

Next, we investigated by two independent approaches, which conserved epitopes act to select cross-reactive GC B cells. First, we used the clonal IgGs from single-cell Nojima culture supernatants for epitope mapping, an approach that allows high-throughput analysis of B cell specificity and avidity^13^. Second, we masked the identified epitopes by injecting specific mAb into mice^16^ and assessed how significantly the epitopes contribute to the selection in vivo.

We screened the clonal IgGs secreted by cross-reactive lung GC B cells and found that 61% (100/163) bound a synthesized peptide encompassing long alpha helix (LAH) in HA2 (Fig. 1b)^17^. In contrast, only two GC B cell clones (1.2%, 2/163) bound head-only HA proteins^14^. Epitopes recognized by the remaining cross-reactive GC B-cell clones (36.8%, 60/163) remain to be identified and characterized in greater details.

An important class of broadly neutralizing HA antibodies recognize conformational stem epitopes, hereafter denoted as “conformational stem (CS)” antibodies. We selected five, well characterized CS mAbs (FI6, MEDI8852, 16.a.26, 31.a.83, and 56.a.09)^18–20^, and assessed their binding to full length rHA. Among these five CS mAbs, we found MEDI8852 to most effectively and specifically compete the other CS mAbs for the binding (Supplementary Fig. 1). We chose, therefore, to identify potential CS antibodies among the cross-reactive clonal IgGs by competition with MEDI8852. Only one (1/163; 0.6%) clonal IgG was effectively competed by MEDI8852 and neither bound the LAH peptide nor HA head-only protein (Supplementary Fig. 1). Summarizing the epitope analysis of HA-binding IgG antibodies from cross-reactive GC B cells, we found 61% to be LAH binders, 1.2% to bind the HA head, 0.6% reactive with a CS epitope; 37% of the GC clonal IgGs were not mapped to known epitopes in our screen and were negative for the three parameters tested.

To confirm the dominancy of LAH epitope among cross-reactive GC B cells, we masked the LAH epitope by injecting mouse LAH mAb (clone mLAH1) into X31-infected mice. We used mAbs with poorly sialylated Fc to avoid Fc-mediated, immune modulation as previously described (Supplementary Fig. 2)^21,22^. Mice were treated with mLAH1 mAb at day 20 after infection, which was the time point when GC B cell numbers plateaued^12^. In support of the epitope mapping data, mLAH1 selectively reduced the number of cross-reactive GC B cells, but it did not affect the numbers of strain-specific GC B cells (Fig. 1c, d). In contrast, passive administration of X31 strain-specific mAb, 1E11, had negligible effects on either GC B cell subset, suggesting that the 1E11 epitope was minor among the hypervariable epitopes for strain-specific GC B cells. Thus, the in vivo epitope masking by antibody infusion confirmed that LAH is the major epitope for selecting cross-reactive GC B cells at the site of viral replication.

### LAH antibodies provide heterosubtypic protection

The LAH amino acid sequences exhibit about 60% homology between H3 and H7 HA subtypes within group 2, but the similarity decreases slightly, to around 50%, between H3 and group 1 HA subtypes (H1 and H5). To address the heterosubtypic reactivity of H3 LAH-binding antibodies, we examined the reactivity of clonal IgGs from individual Nojima cultures against H7 (intra-group) and H1 and H5 (inter-group) HA proteins (Fig. 1e). About one quarter of H3 LAH-binding IgGs also bound the H7 subtype, but fewer recognized H5 (16%) and H1 (3%) proteins.

LAH was originally identified as the epitope of mouse cross-reactive mAb that reactive to drifted H3 but not to H1 HAs^23^. We randomly selected five LAH-binding B cell clones to made recombinant LAH IgG for testing virus neutralizing activity in vitro using homologous X31 as the challenge virus. For reference, we used MEDI8852 and FI6 as representative CS antibodies, which have highly potent, broadly neutralizing activity^18^. Indeed, both CS mAbs showed H3 virus neutralizing activity at 1.6 μg per mL (MEDI8852) and 3.1 μg per mL (FI6), whereas five LAH mAbs did not show any neutralizing activity up to 50 μg per mL under the same condition (Supplementary Fig. 3).

Despite the lack of virus-neutralizing activity under in vitro, there is accumulating evidence for protection provided by non-neutralizing HA mAbs in vivo^24,25^. Therefore, the protective ability of representative LAH mAbs (mLAH1 and mLAH2) of the IgG2a subclass was evaluated in X31-infected mice. Prophylactic treatment with LAH mAbs provided significant protection against lethal infection by X31 based on loss of body weight and survival (Fig. 1f); however, the same LAH IgG2a, bearing N297A mutation, partially reduced the protective function in the same challenge experiment. Since the N297A mutation reduces IgG binding to FcγR^26^, non-neutralizing LAH IgGs appear to provide protection through Fc-mediated effector functions, similarly to the protection provided by CS IgGs^27^, although the levels of Fc dependence are variable among LAH mAb clones. Heterosubtypic protection against H7N9 infection was also observed by the prophylactic treatment of LAH mAbs (Fig. 1g). Together, H3 LAH-binding IgGs are non-neutralizing but broadly protective antibodies, the activity partly dependent on Fc-mediated effector functions.

### LAH-binding memory B cells are present in human

Owing to past infection and/or vaccination histories, almost all healthy adults have pre-existing memory B cells against influenza HA, which significantly affect the recall antibody responses to future infection and vaccination^28^. It is therefore important to know the frequency of the LAH-binding B cells in the human memory compartment. From the peripheral blood mononuclear cells of seronegative donors (hemagglutination inhibition titers were < 40), we identified cross-reactive B cells from human peripheral blood mononuclear cells using a similar, but slightly different, strategy for mouse B cells. First, we used HA probes from a recent H3N2 vaccine strain, A/Victoria/361/2011, because Vic HA-binding memory B cells are considered to be more abundantly present than those against X31 (reassortant virus bearing HA of 1968 strain) and Urg (isolate from 2007) HAs. Second, we collected Vic HA-binding B cells as double positive cells for Vic HA probes that are labeled by different fluorochromes to reduce contamination by unspecific binders. A similar approach has been proven to reduce the contamination of fluorochrome-binding B cells, a significant problem in humans^29^.

**Fig. 2:**
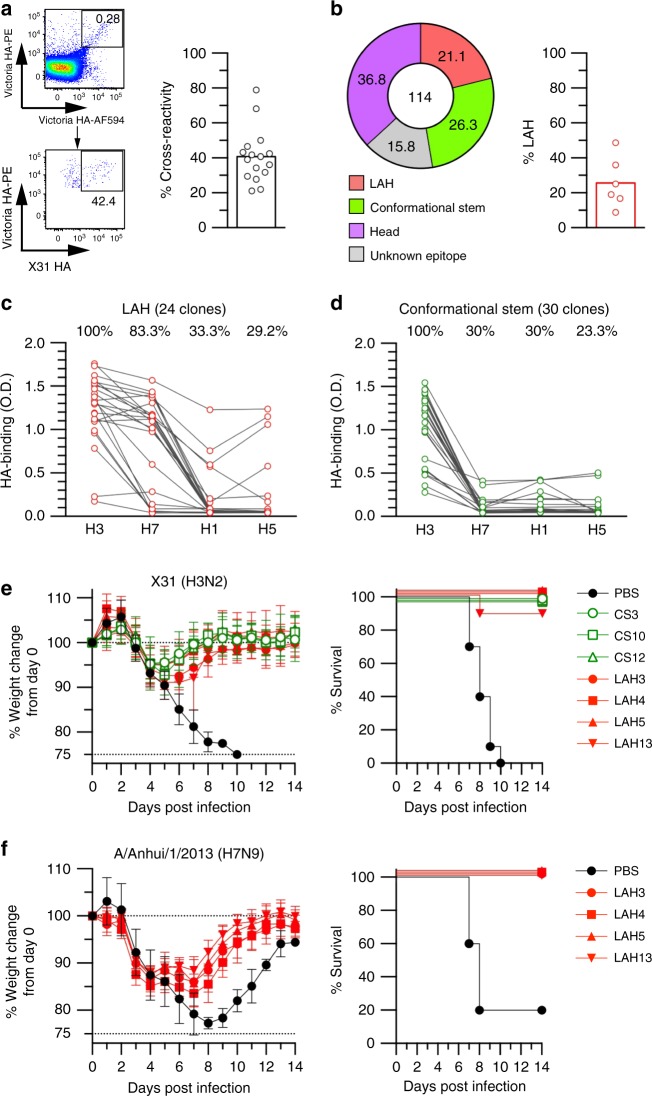
Human LAH-binding B cells are present in cross-reactive memory fraction. **a** Victoria HA-binding IgG^+^ memory B cells (CD19^+^CD27^+^) were identified in human PBMCs as double positive cells (Victoria HAs labeled by AlexaFluor594 and PE) and cross-reactivity to X31 HA was enumerated as gated. Cross-reactivity was expressed as the percentage of X31 HA-binding cells over Victoria HA-binding cells. Each circle represents the result of an individual donor. **b** High-throughput epitope mapping analysis identified multiple classes of conserved epitopes for cross-reactive memory B cells in humans. The data from pooled 6 donors is shown. LAH ratios are shown from individual donor. **c** Heterosubtypic HA reactivity of LAH-binding clones was examined by ELISA using the same rHAs with Fig. 1, except Victoria HA for H3 in Fig. 2. Each dot represents the result from individual clone. **d** Heterosubtypic HA reactivity of CS-binding clones was examined by ELISA under the same condition with **c**. Each dot represents the result from individual clone. **e**, **f** Protective function of CS (green) and LAH (red)-binding IgG2a (100 μg per mouse) was assessed in the mice after lethal dose of homologous H3N2 (X31; 5 x LD50) infection **e** and heterosubtypic H7N9 (A/Anhui/1/2013; 1 x LD50) infection **f**. Body weight (left) and survival (right) of infected mice were monitored daily. The combined data from two independent experiments (*n* = 9–10 per group) are shown. Values represent mean ± s.d. Source data are provided as a Source Data file

We observed that about 40% of the Victoria HA-binding IgG^+^ memory B cells were cross-reactive to X31 HA (Fig. 2a). Single cell cultures confirmed that 75% IgG-secreting clones were HA-binding (Supplementary Table 2). Epitopes of cross-reactive IgG^+^ memory B cells were mapped using the culture supernatants^14^ and LAH binders were identified from 21% clones (Fig. 2b). LAH binders were present from all 6 donors (8.8 to 48.6%), showing the multi-donor presentation of this population (Fig. 2b and Supplementary Table 3). The frequency of LAH binders was lower than that from mouse GC B cells (61%), at least in part because MEDI8852 competitors (CS binding) were abundant in human memory B cells (26%) (Fig. 2b and Supplementary Table 2). We also noticed that 37% of the cross-reactive clones recognized head-only HA proteins, consistent with recent publications in which substantial fraction of the cross-reactive memory B cells/antibodies were head reactive in humans^14,24^.

Breadth of specificity to heterosubtypic HAs was examined in H3 LAH-binding clones in parallel to CS-binding clones that are paradigmatic of broadly neutralizing HA mAbs (Fig. 2c, d). Notably, the majority (83%) of human LAH-binding clones were broadly reactive to H7 and about one third of clones were even bound to H1 and H5 HAs (Fig. 2c). CS-binding clones included broadly reactive clones to H7, H1, and H5 as expected, but the frequencies and the levels of binding on average were lower than those of LAH-binding clones (Fig. 2d).

The functional properties of LAH- and CS-binding mAbs derived from human memory B cells were assessed by in vitro virus neutralization assay and in vivo protection assay using X31 virus. Consistent with previous findings from mouse LAH mAbs, human LAH-binding mAbs (*n* = 4), regardless of the levels of broad reactivity, did not show detectable virus neutralizing activity to X31 in vitro ( > 50 μg per mL), whereas the CS-binding mAbs neutralized with detectable concentration (7.2 ± 5.1 μg per mL, *n* = 5) (Supplementary Fig. 3). Despite the lack of neutralizing activity in vitro, LAH-binding mAbs provided levels of protection similar to those provided by control CS-binding mAbs in vivo at < 5 mg per kg (Fig. 2e). Moreover, LAH-binding mAbs afforded the heterosubtypic protection against lethal infections by avian H7N9 virus strain (Fig. 2f). Taken together, these observations demonstrate that LAH-binding IgG antibodies in humans are broadly protective in vivo, independent of in vitro virus neutralizing activity.

### Conserved epitopes are occluded in native trimeric HA

**Fig. 3:**
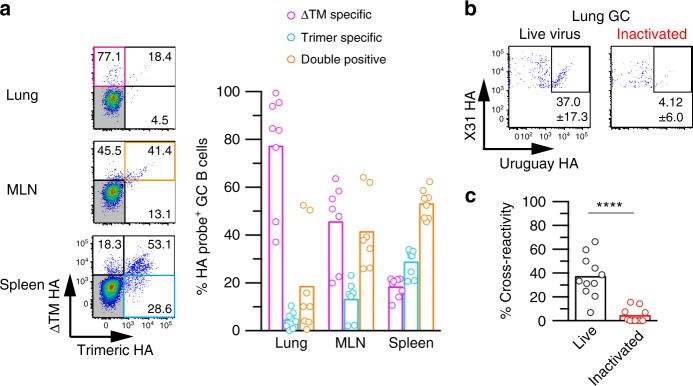
Conserved epitopes are occluded in native trimeric HA. **a** GC B cells were gated on the indicated tissues from X31-infected mice (day 20) and used for the binding assay to ΔTM HA and trimeric HA. The percentage of GC B cells in three fractions (ΔTM-specific, trimer-specific, and double-positive) were quantitated and plotted. Each circle represents the result from an individual mouse. The combined data from two independent experiments are shown. **b** Cross-reactivity was examined from lung GC B cells in the mice inoculated with live X31 virus or inactivated X31 virus. **c** Cross-reactivity was plotted from each group of mice. Each circle represents the result from an individual mouse. The combined data from three independent experiments are shown. The *P*-value was determined with a two-tailed Mann–Whitney test. ****P < 0.0001. Source data are provided as a Source Data file

HA proteins lacking transmembrane domain (ΔTM HA) generates various HA forms including monomeric form^30^. Since we used the ΔTM HA as the probe to detect the HA-binding B cells, the usage of non-native HA as probe might be one technical reason for the successful isolation of LAH-binding B cells. Consequently, we compared B cell access to the HA epitopes present on the ΔTM HA and trimeric HA. For this purpose, we recovered GC B cells from virus-infected mice and analyzed the binding ability of BCRs to both HAs (Fig. 3a and Supplementary Fig. 4). More than half of the splenic GC B cells were categorized into double binders with the binding capabilities comparable to both HA antigens. In contrast, the ratios of double binders were significantly reduced in the mediastinal lymph node and lung GC B cells; reciprocally, ΔTM HA-specific populations increased up to 80% in the lungs where viral replication occurred. Together, these results suggest that lung GC B cells were predominantly selected by the epitopes, which were occluded in native trimeric HA, but were exposed in non-native HA that includes monomeric form.

To assess the requirement of viral replication for the exposure of occluded epitopes, we intranasally inoculated live or inactivated viruses into the mice and induced lung GC B cells by the viruses with or without replication. LAH antibodies bound to inactivated virus, but LAH epitope was apparently exposed at lower effective density than epitopes for strain-specific and CS-binding antibodies (Supplementary Fig. 5). We detected HA-binding lung GC B cells after intranasal injection of inactivated X31 viruses, albeit at lower frequencies than after live virus infection (525.5 ± 512.0 per lung from live virus versus 96.3 ± 74.2 per lung from inactivated virus, *n* > 10). Among the X31 HA-binding GC B cells gated, 37% on average were cross-reactive after live virus inoculation, but only 4% of GC B cells were double binders in mice that received inactivated viruses (Fig. 3b, c). These results demonstrate that the exposure of occluded epitopes is dependent on virus replication.

### LAH epitope is exposed in post-fusion HA

During the viral replication process, influenza viruses convert the native HA into a post-fusion HA form under the acidic conditions within endosome^31,32^. Importantly, the structure of post-fusion HA antigen exposes LAH epitope toward membrane-distal regions, likely reducing the steric hindrance for antibody access that occurs in native, pre-fusion HA^31,32^. Therefore, we tested whether post-fusion HA antigen is the selecting antigen at the site of viral replication through three approaches.

**Fig. 4:**
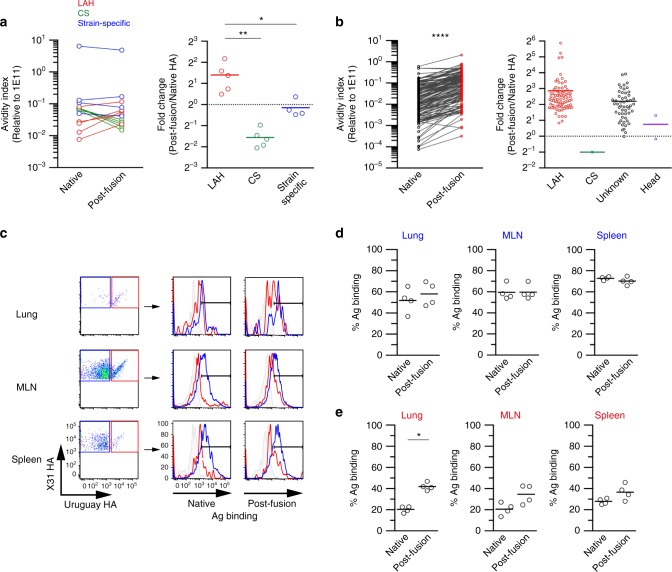
LAH epitope is exposed in post-fusion HA antigen. **a** HA-split antigen (X31) was converted into post-fusion form by treating at pH 5.0. Avidity index toward native and post-fusion HA antigens were determined from purified mAbs (LAH; 5 clones, CS; 5 clones, strain-specific; 4 clones) using strain-specific 1E11 clone as reference. Fold change was calculated by avidity index and comparably plotted from the three groups. Each dot represents the result from individual mAb clone. **b** Avidity index toward native and post-fusion HA antigens and fold change were plotted using the same supernatants from cross-reactive GC B cells in the lungs. **c** Cross-reactive and strain-specific GC B cells were gated, and the percentages of cells binding to native and post-fusion HA antigens that were labeled with fluorochromes were assessed by flow cytometry. Control histogram (gray filled); Narita HA-binding GC B cells of Narita virus-infected mice. **d**, **e** The percentages of strain-specific **d** and cross-reactive cells **e** binding to native and post-fusion HA antigens were plotted. Each circle represents the result from an individual mouse. The representative data from two independent experiments are shown. The *P*-values were determined with a two-tailed Mann–Whitney test (**a**, **e**) and Wilcoxon matched-pairs signed rank test **b**. *P < 0.05; **P < 0.01; ****P < 0.0001. Source data are provided as a Source Data file

First, we assessed the binding of the mAb panel toward native and post-fusion HA antigen by ELISA and calculated the avidity index, as previously described (Fig. 4a)^13^. Strain-specific mAbs bound equally to both forms of the HA antigens, an observation that supports the conservation of strain-specific epitopes in post-fusion HA created by low pH treatment. As expected, post-fusion HA antigen modified the native conformation of CS epitopes and reduced binding by CS mAbs. On the contrary, the binding of LAH-binding mAbs to post-fusion HA antigen was substantially better than to native HA.

Second, we examined the avidity index of a large panel of cross-reactive mAbs from mouse Nojima cultures (Fig. 4b). The comparative analysis of avidity index toward native and post-fusion HA antigens supported that majority of the cross-reactive antibodies, irrespective of LAH- and non-LAH binding, bound the post-fusion HA antigen more avidly than to the native HA antigen. These binding data using soluble form of mAbs demonstrate that the LAH epitope, along with other occluded epitopes, is exposed in post-fusion HA antigen.

Membrane-bound BCRs on the surface of B cells are more susceptible to HA structural constrains than antibodies in soluble form, owing to restricted orientation for HA binding. As the third approach, we assessed the binding ability of membrane-bound BCRs on GC B cells (strain-specific and cross-reactive, separately) toward post-fusion *versus* native, pre-fusion HA antigens by flow cytometry (Fig. 4c). Strain-specific GC B cells from all organs bound equally to both forms of HA antigens (Fig. 4d), supporting again that strain-specific epitopes were relatively conserved in post-fusion HA antigen created by our condition. In contrast, cross-reactive GC B cells bound better to post-fusion HA antigen relative to native HA antigen, and the differential binding was most evident in the lung GC B cells (Fig. 4e). The increased access of cross-reactive GC B cells toward post-fusion HA antigen further strengthens our idea that the selection for lung GC B cells is mediated by post-fusion HA antigen that exposes LAH epitope along with strain-specific epitopes. This also accounts for the equivalent selection of strain-specific and cross-reactive GC B cells in the lungs.

### Post-fusion HA antigen elicits LAH-binding GC B cells

**Fig. 5:**
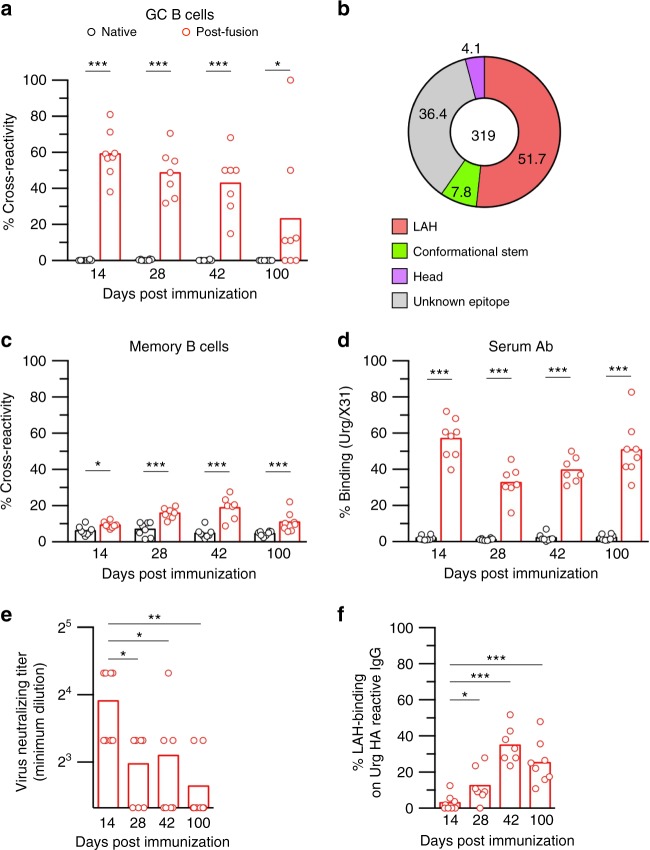
Post-fusion HA antigen elicits LAH-binding GC B cell responses. **a** Native (black) and post-fusion HA (red) antigens with AddaVax adjuvant were i.p. injected into the mice. At the indicated time points, cross-reactivity of HA-binding GC B cells in spleens was enumerated by flow cytometry. **b** High-throughput epitope mapping analysis identified multiple classes of conserved epitopes for cross-reactive GC B cells in mice at day 14 after immunization with post-fusion HA. The data from pooled 9 mice is shown. **c** Cross-reactivity of HA-binding memory B cells were enumerated from the same mice by flow cytometry. **d** IgG titers against X31 HA and Urg HA were determined by ELISA and the binding ratios were plotted. **e** Serially diluted immune sera were subjected to microneutralization assay using Urg strain as challenging virus. **f** Ratios of LAH-binding IgG among Urg HA-binding IgG were evaluated by competitive ELISA. Each dot represents the result of an individual mouse. The combined data from two independent experiments are shown. The *P*-values were determined with a two-tailed Mann–Whitney test. *P < 0.05; **P < 0.01; ***P < 0.001. Source data are provided as a Source Data file

It is technically challenging to isolate the selecting antigens from local GCs and compare the antigenic structure with that of post-fusion HA antigen; therefore, we decided to compare the antibody epitope profiles selected by both antigens. We have already completed the epitope profile from local GC B cells in Fig. 1b, so that we determined the antibody epitope profile in the mice that received the post-fusion HA antigen as an immunogen. HA-binding, splenic GC B cells elicited by the post-fusion HA immunogen were quantitatively comparable to those by the native HA immunogen beyond day 14 after immunization (Supplementary Fig. 6), confirming the equivalent immunogenicity of post-fusion HA antigen. In contrast, the ratios of cross-reactive GC B cells elicited by the two immunogens were totally different; about half of GC B cells elicited by post-fusion HA antigen were double binders as we had previously observed in the local GC B cells (Fig. 1)^12^, whereas such cross-reactive GC B cells was below the detection limit after immunization with native, pre-fusion HA antigen (Fig. 5a). Then, cross-reactive GC B cells were applied to Nojima cultures for determining the contribution of LAH epitope as the selecting epitopes (Fig. 5b and Supplementary Table 4). Through the analysis of 319 cross-reactive GC B cell clones, LAH-binding antibodies were dominant (52%), and CS-binding antibodies and head-binding antibodies were minor (8 and 4%), which reproduces the epitope profile in infection-induced lung GC B cells (Fig. 1b). Thus, the comparable antigenic properties along with the improved access by cross-reactive B cells/antibodies support that post-fusion HA antigen is the selecting antigen in local GCs and eliciting LAH-binding GC B cells at the viral replication site.

GC responses produce at least two types of long-lived B cells: memory B cells and long-lived plasma cells. Analogous to GC responses, the numbers of HA-binding memory B cells were comparable in the mice receiving post-fusion and native HA antigens. However, surprisingly, cross-reactive memory B cells were less frequent than the equivalents in GC B cells up to day 100 after immunization (Fig. 5c). When we extended the analysis to long-lasting serum IgG titers produced by long-lived plasma cells, we noticed that post-fusion HA immunogen robustly induced IgG antibodies that were cross-reactive to drifted Urg HA and LAH peptide (Supplementary Fig. 6). This led to increased frequencies of Urg HA-binding IgG antibodies in the serum, reaching levels comparable with that of GC B cells (Fig. 5d). These data suggest that the altered antigenicity of post-fusion HA antigen prompted the recruitment of cross-reactive B cells into plasma cells rather than into memory B cells. Moreover, we detected low levels of neutralizing activity against drifted Urg virus in day 14 serum (Fig. 5e), but the activity was significantly reduced by day 28 after immunization, even though Urg HA-binding antibody titers were stable or rather enhanced from days 14 to 28 (Supplementary Fig. 6). These data suggest that antibodies neutralizing drifted viruses promptly emerge in the early time points, but they gradually decline with time. In stark contrast to the transient elevation of the cross-neutralizing antibodies, LAH antibodies were minorly represented within the drifted HA-binding IgG fraction in the early time points, but their ratios increased toward day 42, and the numbers were maintained up to day 100 (Fig. 5f). Thus, post-fusion vaccination elicited two waves of cross-reactive antibody responses that were previously unappreciated; prompt and transient induction of neutralizing antibodies in the first wave, and gradual and persistent induction of non-neutralizing LAH antibodies in the second wave.

### Post-fusion HA antigen elicits cross-protective antibodies

**Fig. 6:**
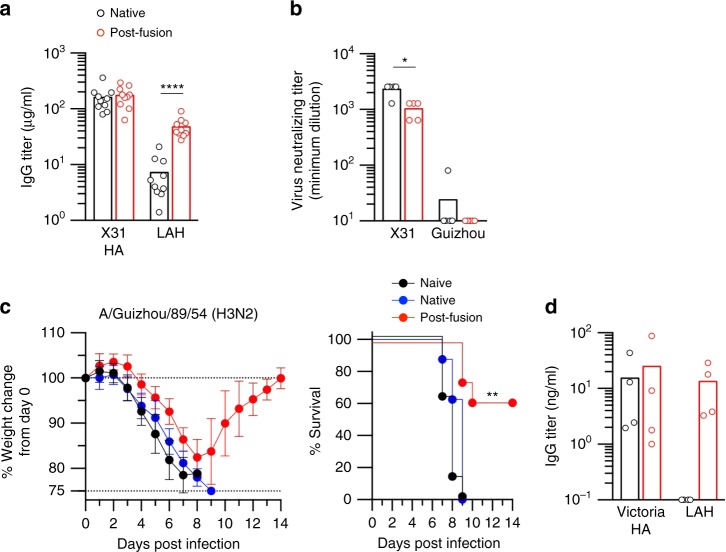
Post-fusion HA antigen elicits protective serum antibodies against drifted virus. **a** Native and post-fusion HA antigens were i.p. injected twice at 4-week intervals. Serum were collected at day 14 after boosting, and subjected to ELISA that detects X31 HA- and LAH-binding IgG. Each circle represents the result from an individual mouse. The combined data from two independent experiments are shown. **b** Immune sera were subjected to a microneutralization assay using H3N2 virus strains (X31 and Guizhou) as challenging viruses. Each circle represents the result from pooled sera (*n* = 5). **c** The protective function of the immune sera (blue, native; red, post-fusion HA) and non-immune sera (black, naive) as control were i.p. transferred into the mice, which were then challenged by lethal dose (5 × LD50) of heterogeneous H3N2 (Guizhou) infection. The combined data from two independent experiments (*n* = 8 per group) are shown. Values represent mean ± s.d. **d** NOJ mice were reconstituted with human PBMCs and then boosted with native and post-fusion HA antigens (Victoria strain). At day 10 post-vaccination, sera were collected and analyzed for anti-HA and anti-LAH human IgG titers by ELISA. Each circle represents the result from an individual mouse harboring PBMC of single donor. The *P*-values were determined with a two-tailed Mann–Whitney test (**a**, **b**) and log-rank test (**c**). *P < 0.05; **P < 0.01; ****P < 0.0001. Source data are provided as a Source Data file

Upon boosting, post-fusion HA antigen induced LAH-binding IgG 7-fold higher than native HA antigen, regardless of comparable HA-binding IgG titers between the two types of HA antigens (Fig. 6a). Using the immune serum, we assessed the functions of polyclonal antibodies elicited by post-fusion HA antigen. First, virus neutralizing activity of the immune serum was examined against the homologous (X31) and heterologous (Guizhou) H3N2 viruses (Fig. 6b), both of which were pathogenic to BALB/c mice and applicable to in vivo challenge experiments in comparison to in vitro assay. The immune serum elicited by both HA antigens exhibited virus neutralizing activity to homologous virus, which was again consistent with the idea that strain-specific epitopes were relatively well-conserved in post-fusion HA antigen. In contrast, neither of the serum exhibited neutralizing activity against heterologous virus. Therefore, cross-neutralizing antibodies were neither boosted nor maintained after boosting by post-fusion HA antigen. We also performed passive transfer experiments to assess in vivo protective function of the same immune serum. It is important to mention that HA-binding antibodies in the immune serum were predominantly IgG1 subclass, which is less protective than IgG2 subclass owing to low affinity binding to activating FcγRs^27^. Moreover, we infused the immune serum containing HA-binding IgG antibodies at < 1 mg per kg body weight of recipient mice. Even under such suboptimal conditions, the immune serum from post-fusion immune mice provided partial but significant protection against the lethal infection by the heterologous strain (Fig. 6c), whereas the immune serum from native HA-immune mice did not provide any protection at all. Thus, post-fusion HA antigen can induce cross-protective IgG antibodies in the serum of vaccinated mice.

To clarify antigenic properties of post-fusion HA antigen on human memory B cells, we used human PBMC-transplanted immunocompromised NOD/SCID/Jak3^-/-^ (NOJ) mice under the conditions where human memory B cells can be restimulated by influenza vaccines^33^. The NOJ mice produced comparable amounts of HA-binding human IgG responses after immunization with two forms of HA antigens (Fig. 6d); however, a majority of human IgG responses were found to recognize LAH epitope after immunization with post-fusion HA antigen only, but not with native HA antigen. Thus, post-fusion HA antigen is able to effectively boost LAH-binding IgG antibodies from human memory lymphocytes, implying the potential application of this immunogen as a vaccine for human use.

## Discussion

Natural influenza virus infections broaden antibody specificity and afford cross-protection against mutant viruses at local infection sites^34,35^. We have found local GCs, ectopically formed at the site of viral replication, as the key induction sites of cross-reactive antibodies that target conserved epitopes^12^. With the introduction of high-throughput single-cell cultures, we identified the LAH epitope as a dominant selecting determinant that is occluded in native HA trimer but exposed in the post-fusion HA antigen as immunogenic epitopes. The antigenic properties of post-fusion HA antigen and its elevated accessibility by cross-reactive B cells/antibodies support the post-fusion HA antigen as the major selecting antigen in local GCs. We have demonstrated a unique property of GC selection at sites of infection that has significant functional relevance for eliciting cross-protective antibodies.

Recent studies revealed the heterogeneity of GC selection that is proposed to arise, at least partly, from epitope modification of selecting antigens in vivo^13,36^. The post-fusion HA antigen provides the example of a non-native antigen, which promotes B cell selection toward conserved epitopes that are occluded, and poorly immunogenic, in the native antigen. Post-fusion HA forms emerges under the acidic conditions (pH 5.0) in endosomes during viral replication. This location raises the question of how the endosomal HA antigen can act as selecting antigen for GC B cells and displayed by follicular dendritic cells (FDCs). In this context, at least two possibilities can be envisaged. First, many influenza-infected cells die; the death process may release the intracellular viral antigens for trapping by FDCs. Second, extensive inflammatory reactions against pathogens may generate extracellular acidification, creating post-fusion HA proteins outside of the cell^37,38^. In either events, we think it is important to note that several protective human HA mAbs recognize occluded epitopes that are exposed in the low pH HA conformation^25,39^. These observations strongly support that the selection by post-fusion HA antigen is not unique to our experimental conditions but rather a commonly observed event in both humans and mice.

We dissected two types of cross-reactive antibodies in temporally separated phases using the mice immunized with post-fusion HA antigen. Cross-neutralizing antibodies arise during the early time points, and non-neutralizing but cross-protective antibodies start to emerge later in the circulation concordantly to the decline of neutralizing antibodies (Fig. 5). We speculate that the early wave of neutralizing antibody development shown in this study represents germline-encoded cross-reactivity through the GC-independent pathway that can be enhanced by rapamycin-mediated mTOR inhibition and GC suppression^6^. In contrast to the previous finding under GC-suppressed condition^6^, the early, neutralizing antibody responses declined over time, suggesting that GC-derived antibodies in the late time points replace the pre-existing neutralizing antibodies by competition. In line with this, H5N1 vaccinee produced cross-reactive (H5/H1) memory B cells against stem domains, but the memory B cells were found to be less persistent than those against head domains in clinical study^40^. Thus, it would be interesting to examine whether neutralizing CS antibodies are less competitive with other HA antibodies in animal model.

An important question remains elusive how non-neutralizing LAH antibodies provide broad protection in vivo. The study using IgG mutant (N297A) in Fig. 1f supports the involvement of Fc-mediated protection, although the levels of Fc dependence are variable among LAH mAb clones. Of note, the similar heterogeneity on Fc dependence was previously shown in human protective IgGs against occluded epitopes that are exposed in low pH-induced HA conformation^25,39^; some of them require Fc-mediated mechanisms for maximizing the protective capacities, but others do not. These data including ours in this study suggest that Fc-mediated pathway contributes to the protection within the limited ranges of antibody dosage and/or their binding properties, such as affinity and epitopes. Once these parameters are under the appropriate conditions, Fc-mediated effector functions, such as antibody-dependent cellular cytotoxicity, antibody-dependent cellular phagocytosis, and/or complement-dependent cytotoxicity may operate and remove the infected cells for viral clearance^39,41–43^. What kind of conditions are required for activating the individual Fc-mediated mechanisms remain to be addressed.

Previous work, including our own, have utilized mouse infection models to demonstrate humoral protection by Fc-dependent pathways^25,27,44–46^. The use of mice, despite the differences in pathophysiology of influenza infection compared to humans, is critical because little information exists on IgG subclasses and FcγR activity, distribution and signaling in more typical animal models (*e.g*., ferret). Indeed, absent this information, any Fc-mediated protection—or lack of it—activity by passively transferred IgGs is difficult to interpret. In this context, we note that a recent clinical study observed a significant correlation of protection with several types of pre-existing antibodies^47^ not only neutralizing HA antibodies but also poorly neutralizing HA and NA antibodies that require Fc-mediated mechanisms to maximize the protection in mice^27,46^. Thus, the limited clinical data suggests the possible relevance of Fc-mediated protection during natural influenza infection in humans.

Elicitation of transient neutralizing antibodies and persistent non-neutralizing antibodies by post-fusion HA antigen provides several important implications for future strategies of universal influenza vaccine. Cross-neutralizing antibodies are important and promising targets that need to be elicited robustly and durably by vaccination. However, the factors hampering the durable elicitation, such as the polyreactivity of CS antibodies^28,48^, need to be further evaluated. Future comparative studies between neutralizing CS and non-neutralizing LAH antibodies might reveal the functional compartmentalization of these two classes of cross-protective antibodies, which would be required for the rational design of a universal influenza vaccine that potentiates broad protection through multiple points of action.

## Methods

### Virus and vaccine

IVR-165 (A/Victoria/361/2011; H3N2) was provided by Commonwealth Serum Laboratories. A/Anhui/1/2013 (H7N9) virus was provided by China CDC. All viruses including X31 (H3N2), A/Uruguay/716/07 (H3N2), A/Narita/1/09 (H1N1), and A/Guizhou/54/89 (H3N2) strains were propagated in embryonated chicken eggs and purified by a sucrose gradient (10–50%) ultracentrifugation. For inactivation, purified viruses were treated with 0.05% formalin at 4 °C for a week. To produce the HA-split vaccines, purified live viruses were suspended in 0.1% Tween80 and mixed with an equal volume of ether. After stirring, the mixture was centrifuged to separate the aqueous and ether phases, and the ether phase was discarded. After several rounds of extraction, the remaining aqueous phase was collected as the HA-split vaccine. For post-fusion HA-split vaccine, HA-split vaccines were mixed with 0.15 M citrate buffer and treated with acidic buffer (pH 5.0) for 30 min to cause irreversible transition into post-fusion form^32^, and then neutralized by adding 1 M Tris–HCl buffer pH 8.0. HA-split vaccines were enriched by ultrafiltration (Sartorius) and then treated with formalin at 4 °C for a week before use.

### Mice

BALB/c mice were purchased from Japan SLC. NOD/SCID/JAK3^-/-^ mice were kindly provided by Dr. S. Okada (Kumamoto University). All mice were maintained under SPF condition and used at 7–12 weeks of ages.

### Virus infection and challenge experiments

BALB/c mice were anesthetized by i.p. (intraperitoneal) injection with sodium pentobarbital and then i.n. (intranasal) infected with X31 virus or Narita virus at a dose of 0.2 × LD50 in a volume of 20 μL. Some groups of the mice were i.p. treated with 100 μg of mAb or 200 μl of immune serum more than 3 h before being challenged by either X31 (5 × LD50), A/Guizhou/54/89 (5 × LD50), or A/Anhui/1/2013 (1 × LD50). All mice were monitored daily for survival and body weight loss until 14 days after infection. The humane endpoint was set at 25% body weight loss relative to the initial body weight at the time of infection.

### Immunization

BALB/c mice were i.p. immunized with native- or post-fusion HA-split antigens (10 μg) along with the adjuvant AddaVax (InvivoGen), and boosted with the same dose of antigens without AddaVax at day 28 after immunization. Sera were collected at day 14 after boosting. In some experiments, mice received i.n. administration of inactivated viruses twice at 28-day intervals, and the lungs were recovered at day 7 after boosting for flow cytometric analysis.

### Humanized mouse assay

Heparinized peripheral blood was obtained from healthy donors. PBMCs were isolated by density centrifugation using Lymphocyte Separation Medium 1077 (PromoCell). Two mice were i.v. injected with 2 × 10^7^ PBMCs from each donor, and then i.v. immunized with 50 μg HA-split antigen 24 h later. The immune sera were collected at day 10 after immunization and subjected to ELISA.

### Murine cell preparation

Mice were perfused with PBS in the right ventricle to clear the blood from lungs. Lungs were minced and incubated at 37 °C for 45–60 min in DMEM containing 5% FBS, 2 mM L-glutamine, 100 IU per mL penicillin, 100 μg per mL streptomycin, 2 mg per mL collagenase D (Roche), and 10 μg per mL DNase I (Roche) and then disrupted between the frosted ends of glass slides. After centrifugation in a 70%/44%/30% Percoll gradient, the cells at the interface between 70% and 44% were recovered for lung cells. MLN or spleen were mechanically disrupted between the frosted ends of glass slides in DMEM containing 2% FBS, 2 mM L-glutamine, 100 IU per mL penicillin, 100 μg per mL streptomycin, and 55 μM 2-mercaptoethanol. For spleens, red blood cells were lysed in ammonium-chloride-potassium buffer (0.16 M NH_4_CL, 10 mM KHCO_3_, and 0.1 mM EDTA) for 1 min and the cells were washed.

### Flow cytometric analysis

Murine cells were pretreated with anti-FcγRII/III mAb, and then incubated with mixtures of biotinylated mAbs for dumping gates; anti-IgM (eBioscience, Cat#: 13-5790-85, 1:1000), IgD (eBioscience, Cat#: 13-5993-85, 1:1000), Gr-1 (eBioscience, Cat#: 13-5931-85, 1:2000), CD3 (BioLegend, Cat#: 100304, 1:1000), CD5 (eBioscience, Cat#: 13-0051-85, 1:5000), CD11b (BioLegend, Cat#: 101204, 1:2000), CD11c (BioLegend, Cat#: 117304, 1:1000), CD43 (BD Biosciences, Cat#: 553269, 1:5000), CD90 (eBioscience, Cat#: 13-0903-85, 1:3000), CD93 (eBioscience, Cat#: 13-5892-85, 1:1000), TER-119 (BioLegend, Cat#: 116204, 1:2000), F4/80 (BioLegend, Cat#: 123106, 1:2000), CD117 (BioLegend, Cat#: 105804, 1:5000), and CD138 (BD Biosciences, Cat#: 553713, 1:1000). The secondary staining was done by a mixture of fluorochrome-conjugated antibodies [anti-CD38 (BioLegend, Cat#: 102720, 1:500), B220 (BioLegend, Cat#: 103232, 1:100)], HA probes, HA-split antigens, streptavidin (BioLegend, Cat#: 405233, 1:200, or BD Biosciences, Cat#: 562284, 1:3000), and propidium iodide (PI) or Live/Dead Fixable Aqua (Life Technologies). The cells were treated with 15 mU neuraminidase (Roche) for 10 min at 37 °C when HA-split antigens were used as probes. In experiments using trimeric HAs as probes, cells were stained with FITC-conjugated mAbs for dumping gate [anti-IgM (BioLegend, Cat#: 406506, 1:200, and eBioscience, Cat#: 11-5790-85, 1:200), IgD (BioLegend, Cat#: 405704, 1:500), CD5 (BioLegend, Cat#: 100606, 1:500), CD11b (BioLegend, Cat#: 101206, 1:500), and CD43 (BioLegend, Cat#: 143203, 1:500)].

For staining of human PBMCs, cells were incubated with mixtures of biotinylated mAbs [anti-IgD (BD Biosciences, Cat#: 555777, 1:400), CD2 (BioLegend, Cat#: 300204, 1:400), and CD4 (BioLegend, Cat#: 300504, 1:400)] and then stained with anti-CD19 (BioLegend, Cat#: 302240, 1:100), CD27 (BioLegend, Cat#: 302814, 1:100), IgG (BD Biosciences, Cat#: 555786, 1:50), HA probes, streptavidin (BioLegend, Cat#: 405233, 1:200), and Live/Dead Fixable Aqua. B cells were sorted from MACS-enriched populations using streptavidin-microbeads (Miltenyi Biotech). Stained cells were analyzed or single-cell sorted using FACS Canto II or FACS Aria III (BD Bioscience). Flow cytometry data were analyzed with the FlowJo software (TreeStar). ΔTM recombinant HA (rHA)^12^ was conjugated with fluorochromes [APC and PE (Dojindo), or Alexafluor594 (Invitrogen)] in our laboratory. Trimeric HA^49^ was labeled with streptavidin-PE (Invitrogen) by sequential addition manner. X31 native- and post-fusion HA-split antigens were labeled with AlexaFluor488 (Invitrogen) in our laboratory.

### Single cell culture of mouse/human B cells

Feeder line cells (NB21.2D9 cells for murine cells^13^ and MS40L-low cells for human cells^14^) were seeded into 96-well plate at 1000 NB21.2D9 cells per well or 3000 MS40L-low cells per well in B cell media (BCM; RPMI-1640 containing 10% Hyclone FBS, 1 mM sodium pyruvate, 10 mM HEPES, MEM nonessential amino acid, 100 IU per mL penicillin, 100 μg per mL streptomycin, and 55 μM 2-mercaptoethanol) one day before single B-cell sorting. Next day, recombinant cytokines [murine IL-4 (Peprotech: 2 ng per mL) for mouse B cells, or human IL-2 (Peprotech: 50 ng per mL), human IL-4 (Peprotech: 10 ng per mL), human IL-21 (Peprotech: 10 ng per mL), and human BAFF (Peprotech: 10 ng per mL) for human B cells] in BCM were added to the cultures, mouse/human B cells were directly sorted into 96-well plates at 1 cell per well, and then co-cultured with feeder line cells. After cultivation, culture supernatants were harvested and subjected to ELISA for mapping the epitopes of the secreted mAbs. Cultured clonal B cells were also frozen for V(D)J sequence analysis and V(D)J gene recovery for making recombinant mAbs.

### ELISA and epitope mapping

For the epitope mapping of single cell culture supernatants, we performed ELISA. ELISA plates were coated with either anti-mouse/human IgG (Fab specific) Ab (Sigma-Aldrich, Cat#: M6898 or I2136, 5 μg per mL), X31 trimeric full-length/head-only rHA^14^, Narita rHA, Victoria rHA, NIBRG-14 rHA, Anhui rHA, LAH peptide, or inactivated X31 virus. In some experiments, rHA was trapped by ELISA plate through plate-bound anti-His-tag Ab (MBL Life Science, Cat#: PM032, 2.5 μg per mL). To determine the binding to HA-split vaccine, non-fixed HA-split vaccine were coated to the ELISA plates, and then treated with/without acidic buffer (pH 5.0) for 30 min. After blocking with PBS containing 1% BSA, culture supernatants were applied to plates at a 1:10, 1:50, 1:150, 1:450, 1:500 or 1:1500 dilution, and then incubated with goat anti-mouse IgG-HRP (SouthernBiotech, Cat#: 1030-05, 1:5000) or goat anti-human IgG-HRP (Southern Biotech, Cat#: 2040-05, 1:5000). For detecting the CS antibodies, MEDI8852 (20 μg per mL) was added to plates before loading the supernatant and then incubated for 2 h. HRP-activity was visualized with OPD substrate (Sigma), and OD_490_ was measured using iMark Microplate Reader (Bio-Rad).

To determine the binding of total IgG and antigen-specific IgG, the threshold OD was set at the point representing the average plus six SDs from negative control wells without sorted B cells. The threshold OD for MEDI8852-competitive ELISA was set at the point representing the average minus three SDs from values of the LAH-positive wells. To obtain the avidity index, the concentrations of total IgG and HA-split-binding IgG were determined with reference to the standard mAb (clone 1E11). The avidity index for HA-split vaccines were obtained by calculating the ratios of antigen-specific IgG/total IgG of each sample. The concentrations of antigen-specific IgG in the serum were determined by ELISA using rHA and the LAH peptide as coating antigens. To determine the ratio of LAH-binding IgG among Urg rHA-binding IgG, the serum was pre-incubated with 10 μg per mL LAH peptide before loaded onto Urg rHA-coated plate.

### Western and lectin blotting of IgG Fc

ACC4 hybridoma secreting mouse anti-citrullinated protein antibody (ACPA) was provided by R. Holmdahl (Lund University). Sialylated ACPA (ACC4 Sia^+^) was constructed by transfecting both *mSt6gal1* and *mB4galt1* cDNAs into ACC4 hybridoma^22^. Purified IgGs were incubated in digestion buffer [10 mM EDTA, 10 mM cysteine in PBS (pH 7.4)] with papain (Sigma-Aldrich, 0.05 μg per 1 μg of IgG.) for 2 h at 37 °C. Digested IgGs were separated by SDS–PAGE (IgGs, 1 μg per lane for lectin blotting; 100 ng per lane for western blotting), and transferred onto Immobilon^®^-P Transfer Membranes (Merck Millipore). For western or lectin blotting, the membranes were incubated with either goat anti-mouse IgG-HRP (Cell Signaling Technology Japan, 1:1000) or biotin-conjugated lectins (Vector Laboratories, Inc.). Terminal α2,6 sialic acid (Sia), β1,4 galactose (Gal) and N-acetylglucosamine (GlcNAc) were detected by SNA (Cat#: B-1305, 1:400), ECL (Cat#: B-1145, 1:1000), and GSL II (Cat#: B-1215, 1:400) lectin, respectively. Biotin-conjugated lectins blots were incubated with ABC kit (Vector Laboratories). Bound conjugates on the membrane were visualized with an Enhanced Chemiluminescence^TM^ detection system (PerkinElmer Life Sciences). Chemiluminescence was detected by Amersham Imager 680 (GE Healthcare).

### In vitro neutralizing assay

Virus-neutralization antibody titers were determined by microneutralization using MDCK cell line (Influenza Virus Research Center, National Institute of Infectious Diseases, Japan). Serially diluted serum or mAb was pre-incubated with either X31, A/Uruguay/716/07, or A/Guizhou/54/89 (100 TCID50), and then added to MDCK cells in the presence of 5 μg per mL acetyltrypsin (Sigma). After 3 days of incubation, the virus-neutralization antibody titer was determined and expressed as the minimal Ab concentrations that inhibit viral replication.

### BCR sequence analysis

Rearranged V(D)J sequence analysis of single-cell cultured mouse/human B cells was performed as follows. Total RNA was extracted from frozen cells after single-cell cultures using RNeasy Plus Micro Kit (Qiagen) and subjected to reverse transcription using SuperScript III CellsDirect cDNA Synthesis kit (Invitrogen) and random hexamer for cDNA synthesis. V_H_ and V_L_ genes were amplified by two rounds of nested PCR with established primers and methods^50,51^. The V(D)J rearrangement and the number of somatic hypermutations were identified using IgBlast IMGT.

### Generation of mAbs

Chimeric mAbs as a form of human/mouse V(D)J with constant regions of mouse IgG2a wild-type or N297A-mutant were generated as follows. The V_H_/V_L_ genes of selected B cell samples from single-cell cultures or published Ab (FI6, MEDI8852, 16.a.26, 31.a.83, and 56.a.09) were cloned into mouse IgG2a wild-type or N297A-mutant expression vectors and then subsequently produced by transfection of Expi293F cells (Thermofisher, Cat#: A14527)^50^. mAbs were purified from the culture supernatant using a protein G column (Thermofisher) and used for further analysis.

### Statistics

Statistical analyses were performed with a two-tailed Mann–Whitney test or Wilcoxon matched-pairs signed rank test. Statistical significance between the survival rates was determined by Kaplan–Meier survival curves and log-rank test. All statistical analyses were performed using Prism (GraphPad). *P*-values < 0.05 were considered significant and indicated by asterisks: *P < 0.05; **P < 0.01; ***P < 0.001; ****P < 0.0001.

### Study approval

The studies using human samples were approved by the Institutional Ethics Committee of Human Experimentation and performed in accordance with the Ethical Guidelines for Medical and Health Research Involving Human Subjects in Japan. All participants provided written informed consent in accordance with the Declaration of Helsinki. Animal procedures were approved by the Animal Ethics Committee of the National Institute of Infectious Diseases, Japan, and performed in accordance with the guidelines of the Institutional Animal Care and Use Committee.

### Reporting summary

Further information on research design is available in the Nature Research Reporting Summary linked to this article.

## Supplementary information

Supplementary Information

Reporting Summary

Source Data

## Data Availability

Complete sequence data that support the findings of this study have been deposited in the DNA Data Bank of Japan (DDBJ), the EMBL Nucleotide Sequence Database, and GenBank under the following accession numbers (LC457978-LC457997). Other data that support the findings of this study are available from the corresponding author upon reasonable request. The source data underlying Figs. 1–6, Supplementary Figs. 1–3, and Supplementary Tables 1–4, are provided as a Source Data file.
