# Supplementary material for: Exposure of an occluded hemagglutinin epitope drives selection of a class of cross-protective influenza antibodies
Source: Nat Commun. 2019 Aug 28;10:3883. doi: 10.1038/s41467-019-11821-6 (PMC6713747; doi:10.1038/s41467-019-11821-6)
Supplement: Supplementary file 1 — Supplementary Information [file 41467_2019_11821_MOESM1_ESM.pdf]

Supplementary Information

Exposure of an occluded hemagglutinin epitope drives selection of a class of cross-protective influenza antibodies

Adachi et al.

Supplementary Figure 1

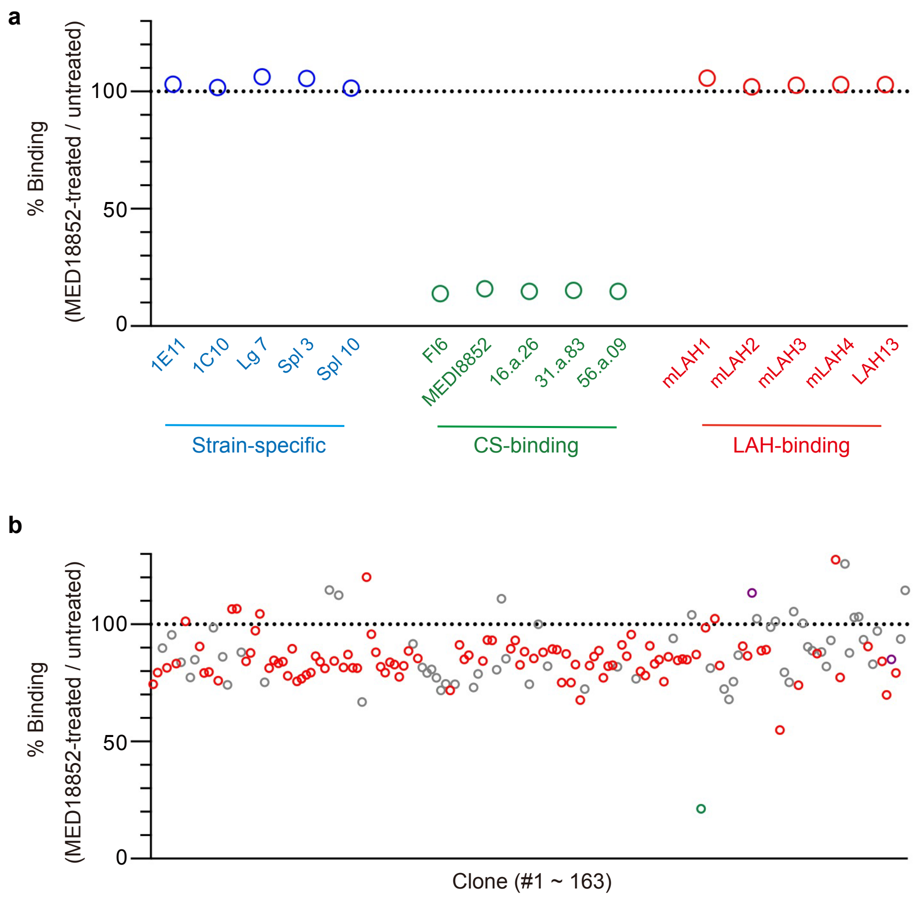

Supplementary Figure 1. Competitive ELISA using MEDI8852 permits the identification of CS-binding mAbs.

a Three groups of mAbs were applied to competitive ELISA using MEDI8852 for confirming the specificity. The percentages of binding were plotted in y axis. Each circle represents the result from individual clone. **b** CS-binding clones were screened from the culture supernatants of 163 clones. Each circle represents the result from individual clone. Red; LAH, green; conformational stem, gray; unknown, purple; head. Source data are providied as a Source Data file.

Supplementary Figure 2

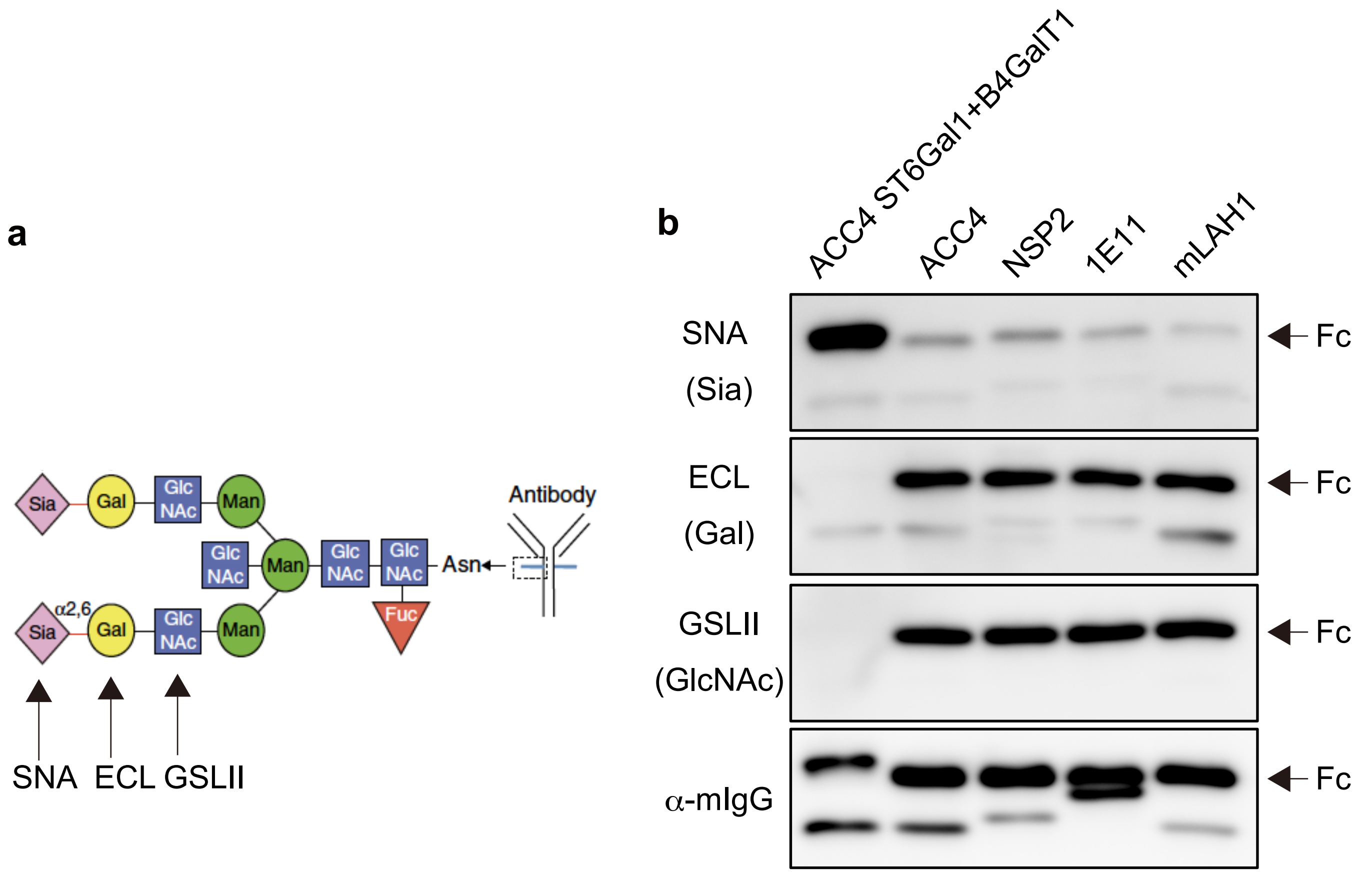

Supplementary Figure 2. The levels of IgG Fc sialylation was evaluated by lectin blotting. **a** IgG Fc at the position of Asn297 is heterogenously glycosylated with either terminal sialic acids or others. IgG Fc with terminal sialic acid (Sia) is detected by SNA lectin and those with Gal and GlcNAc are detected by ECL lectin and GSLII lectin, respectively. **b** The levels of IgG Fc glycosylation was evaluated by SNA, ECL, and GSLII lectin blotting. Highly sialylated ACC4 mAb (ACC4 ST6Gal1+B4GalT1) and normal ACC4 mAb were loaded as controls. The equivalent amounts (1 μg per lane for lectin blotting and 0.1 μg per lane for IgG) of NSP2 (anti-H1 head), 1E11 (anti-H3 head), and mLAH1 (H3 LAH) were loaded after papain cut. Source data are providied as a Source Data file.

Supplementary Figure 3

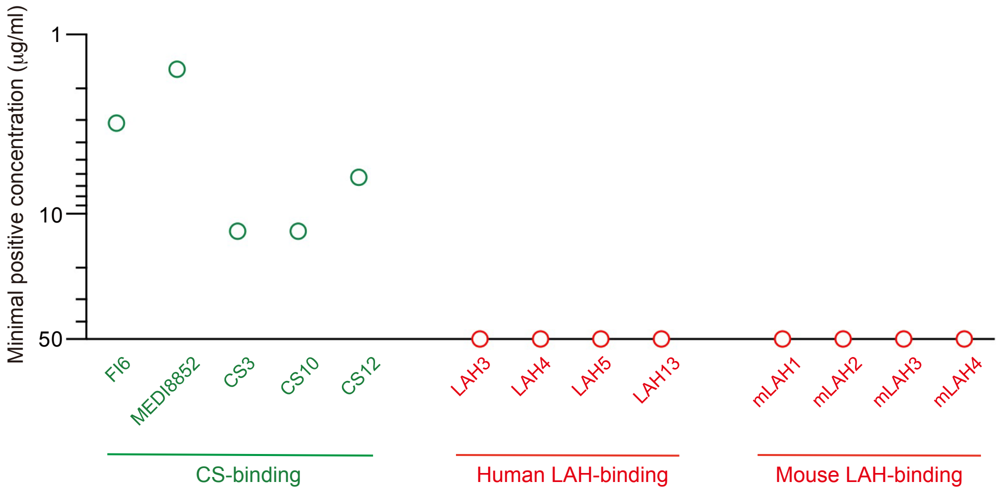

Supplementary Figure 3. Neutralizing activity of mouse and human mAbs to H3N2 virus. CS-binding and LAH-binding mAbs were subjected to *in vitro* neutralizing assay using X31 virus. Each circle represents the result from individual clone. Source data are providied as a Source Data file.

Supplementary Figure 4

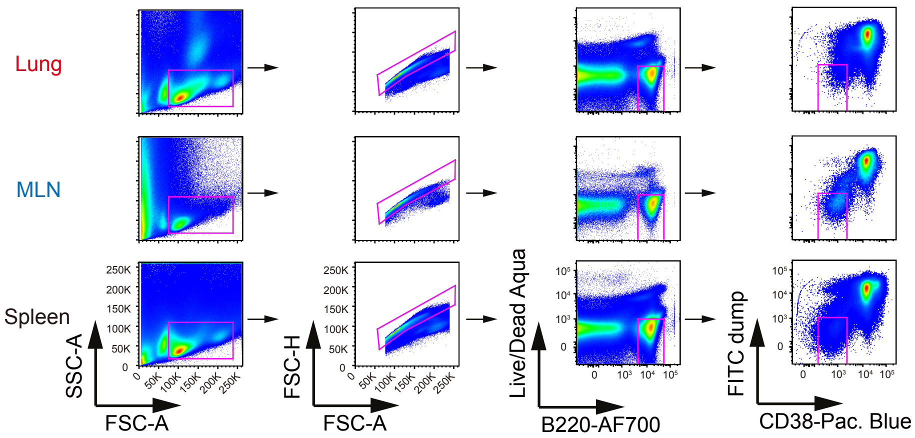

Supplementary Figure 4. FACS gating strategy for GC B cells using trimeric HA probe. Cells were recovered from lung, MLN, and spleen in X31-infected mice at day 20. After lymphocyte gating and selection of singlet cells, $CD38^{dull}$FITC dump (IgM, IgD, CD5, CD11b, CD43)$^{-}$ cells among live $B220^{+}$ fractions were gated for further analysis.

Supplementary Figure 5

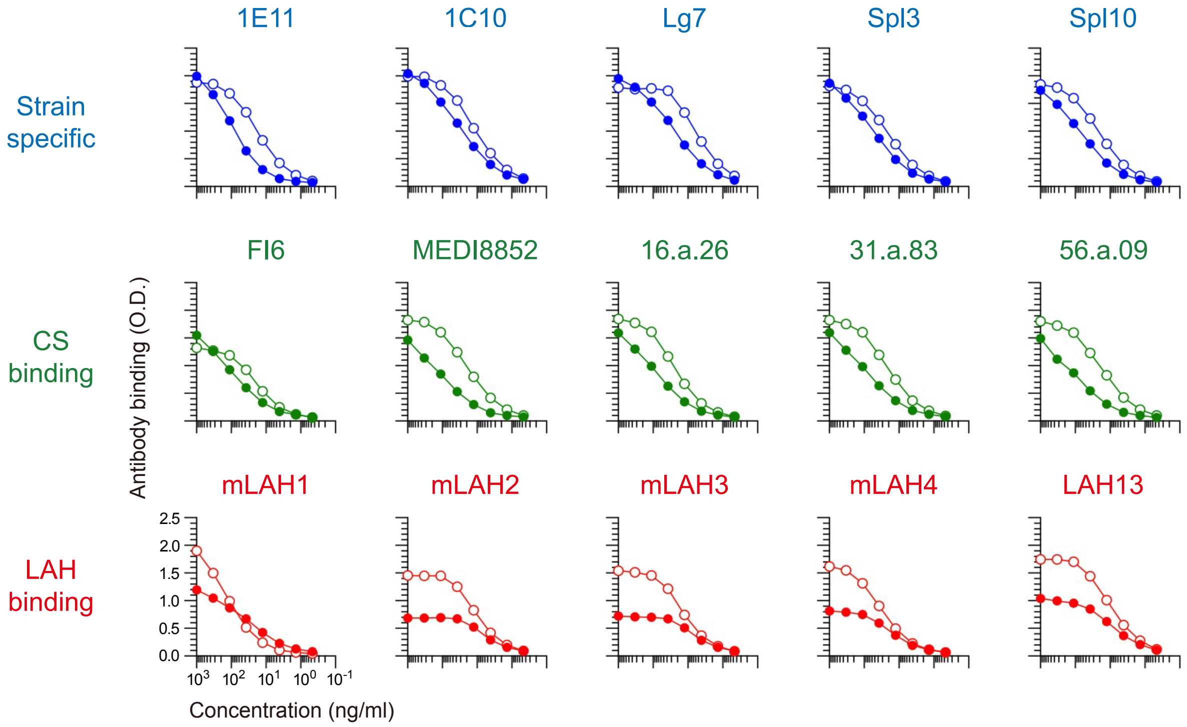

Supplementary Figure 5. Binding ability of LAH mAbs to inactivated virus.
Binding of strain-specific, CS-binding, and LAH-binding mAbs (5 clones from each group) againt inactivated virus was assessed by ELISA (filled circles). The binding to ΔTM HA was also comparably analyzed as reference (open circles). Source data are providied as a Source Data file.

Supplementary Figure 6

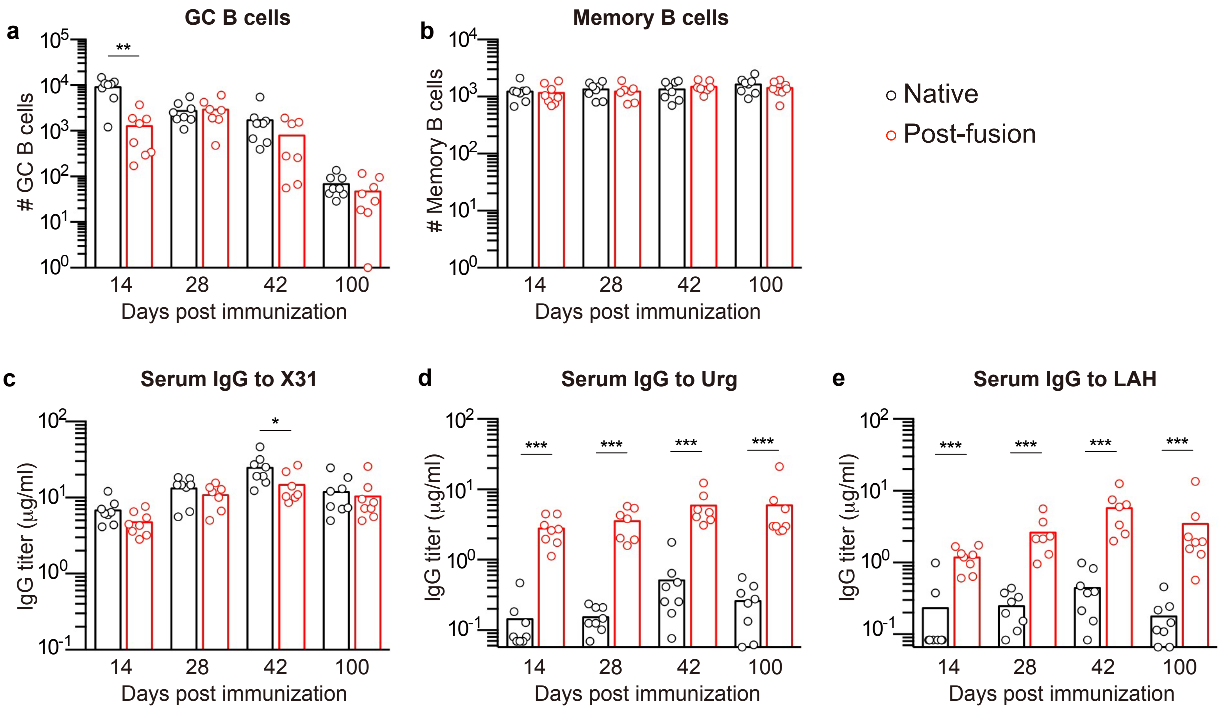

Supplementary Figure 6. GC/memory B cell responses and serum IgG titers elicited by post-fusion HA antigen.

a, b Native HA (black) and post-fusion HA (red) antigens adjuvanted with Addavax were i.p. injected into the mice. At the indicated time points, the numbers of HA-binding splenic GC (**a**) and memory (**b**) B cells were enumerated by flow cytometry. **c-e** Serum IgG titers against X31 HA (**c**), Urg HA (**d**), and LAH (**e**) were analyzed at the indicated time points by ELISA using LAH13 as standard. Each dot represents the result from individual mouse (n = 7-8 per each time point). *, P < 0.05; **, P < 0.01; ***, P < 0.001. Source data are providied as a Source Data file.

Supplementary Table 1

Supplementary Table 1. Summary of mouse single cell cultures for cross-reactive GC B cells in lungs from infected mice.

	Cross-reactive GC B cells
IgG+ / total plated	191 / 1290 (14.8%)
HA-binding IgG	163 / 191 (85.3%)
Head-binding	2 / 163 (1.2%)
Head non-binding	161 / 163
LAH-binding	100 / 163 (61.3%)
Conformational stem	1 / 163 (0.6%)

Supplementary Table 2

Supplementary Table 2. Summary of human single cell cultures for cross-reactive memory B cells.

	Cross-reactive MBCs
IgG+ / total plated	152 / 374 (40.6%)
HA-binding IgG	114 / 152 (75.0%)
Head-binding	42 / 114 (36.8%)
Head non-binding	72 / 114
LAH-binding	24 / 114 (21.1%)
Conformational stem	30 / 114 (26.3%)

Supplementary Table 3

Supplemental Table 3. Summary of human single cell cultures for cross-reactive memory B cells from different donors.

Donor ID	% IgG+ memory B	% Vic HA+	% CR	Total plated	IgG+	HA-binding IgG	LAH-binding	% LAH-binding	HI titer
# 33	26.8	0.147	55.2	380	196	175	85	48.6	< 10
# 40	34.2	0.179	49.8	123	76	58	11	19.0	< 10
# 48	16.8	0.277	79.3	90	44	24	6	25.0	< 10
# 53	21.0	0.203	54.9	197	41	30	5	16.7	< 10
# 60	26.0	0.212	83.0	258	87	57	5	8.8	20
# 61	9.5	0.161	68.8	78	33	25	9	36.0	10
**Total**				**1126**	**477**	**369**	**121**		
							**Average**	**25.7**	
							**S.D.**	**13.2**	

Supplementary Table 4

Supplemental Table 4. Summary of mouse single cell cultures for cross-reactive splenic GC B cells from post-fusion HA immune mice.

	Cross-reactive GC B cells
IgG+ / total plated	363 / 1330 (27.3%)
HA-binding IgG	319 / 363 (87.9%)
Head-binding	13 / 319 (4.1%)
Head non-binding	306 / 319
LAH-binding	165 / 319 (51.7.%)
Conformational stem	25 / 319 (7.8%)
