# Supplementary material for: Exposure of an occluded hemagglutinin epitope drives selection of a class of cross-protective influenza antibodies
Source: Nat Commun. 2019 Aug 28;10:3883. doi: 10.1038/s41467-019-11821-6 (PMC6713747; doi:10.1038/s41467-019-11821-6)
Supplement: Supplementary file 2 — Reporting Summary [file 41467_2019_11821_MOESM2_ESM.pdf]

nature research

Corresponding author(s): Yohimasa TAKAHASHI

Last updated by author(s): 2019-7-29

Reporting Summary

Nature Research wishes to improve the reproducibility of the work that we publish. This form provides structure for consistency and transparency in reporting. For further information on Nature Research policies, see Authors & Referees and the Editorial Policy Checklist.

Statistics

For all statistical analyses, confirm that the following items are present in the figure legend, table legend, main text, or Methods section.

n/a	Confirmed	
☐	☒	The exact sample size (*n*) for each experimental group/condition, given as a discrete number and unit of measurement
☐	☒	A statement on whether measurements were taken from distinct samples or whether the same sample was measured repeatedly
☐	☒	The statistical test(s) used AND whether they are one- or two-sided<br>*Only common tests should be described solely by name; describe more complex techniques in the Methods section.*
☒	☐	A description of all covariates tested
☒	☐	A description of any assumptions or corrections, such as tests of normality and adjustment for multiple comparisons
☐	☒	A full description of the statistical parameters including central tendency (e.g. means) or other basic estimates (e.g. regression coefficient) AND variation (e.g. standard deviation) or associated estimates of uncertainty (e.g. confidence intervals)
☒	☐	For null hypothesis testing, the test statistic (e.g. *F*, *t*, *r*) with confidence intervals, effect sizes, degrees of freedom and *P* value noted<br>*Give P values as exact values whenever suitable.*
☒	☐	For Bayesian analysis, information on the choice of priors and Markov chain Monte Carlo settings
☒	☐	For hierarchical and complex designs, identification of the appropriate level for tests and full reporting of outcomes
☒	☐	Estimates of effect sizes (e.g. Cohen's *d*, Pearson's *r*), indicating how they were calculated

Our web collection on statistics for biologists contains articles on many of the points above.

Software and code

Policy information about availability of computer code

Data collection	FACS Diva software version 7 (BD)<br>Microplate Manager Software 6 (Bio-Rad)
Data analysis	Flowjo version 9.9.6 (Tree Star Inc.)<br>GraphPad Prism version 6 (GraphPad Software Inc.)<br>Microsoft Excel (Microsoft)

For manuscripts utilizing custom algorithms or software that are central to the research but not yet described in published literature, software must be made available to editors/reviewers. We strongly encourage code deposition in a community repository (e.g. GitHub). See the Nature Research guidelines for submitting code & software for further information.

Data

Policy information about availability of data

All manuscripts must include a data availability statement. This statement should provide the following information, where applicable:

- Accession codes, unique identifiers, or web links for publicly available datasets
- A list of figures that have associated raw data
- A description of any restrictions on data availability

Complete sequence data are available from the DNA Data Bank of Japan (DDBJ), the EMBL Nucleotide Sequence Database, and GenBank under the following accession numbers (LC457978-LC457997).

Field-specific reporting

Please select the one below that is the best fit for your research. If you are not sure, read the appropriate sections before making your selection.

☒ Life sciences ☐ Behavioural & social sciences ☐ Ecological, evolutionary & environmental sciences

For a reference copy of the document with all sections, see nature.com/documents/nr-reporting-summary-flat.pdf

Life sciences study design

All studies must disclose on these points even when the disclosure is negative.

Sample size	Sample size was determined accordingly to previous published studies. The sample size takes into account expected attrition (due to death of animals) based on our previous studies using the same virus strains for challenges. All experiments were performed with a minimum of 4 samples per experimental group, and virus challenge experiments were performed by 8-10 mice per group.
Data exclusions	For single B cell culture and epitope mapping, we excluded the samples with IgG titers below the threshold OD. The detailed information was described in ELISA and epitope mapping section of Methods, and the results of single cell culture including frequency of IgG+ well were listed in Supplementary table 1, 2, 3 and 4.<br>For calculation of the avidity index in Figure 4, we excluded 30 samples with insufficient amount of total IgG/antigen-specific IgG in reference to standard mAb.
Replication	The experiments were repeated at a minimum of 2 times. The detailed information was described in each figure legend.
Randomization	Mice were assigned to each experimental groups randomly.
Blinding	The experiments were not blinded; however, data was collected to minimize the observer bias as much as we can. For example, all gating for flow cytometry analyses were performed on control samples before being applied to test samples to minimize biased gating.Investigators were not blinded to group allocation since data collection for each experiments were performed individually.

Reporting for specific materials, systems and methods

We require information from authors about some types of materials, experimental systems and methods used in many studies. Here, indicate whether each material, system or method listed is relevant to your study. If you are not sure if a list item applies to your research, read the appropriate section before selecting a response.

Materials & experimental systems

n/a	Involved in the study	
☐	☒	Antibodies
☐	☒	Eukaryotic cell lines
☒	☐	Palaeontology
☐	☒	Animals and other organisms
☐	☒	Human research participants
☒	☐	Clinical data

Methods

n/a	Involved in the study	
☒	☐	ChIP-seq
☐	☒	Flow cytometry
☒	☐	MRI-based neuroimaging

Antibodies

Antibodies used

Biotin Anti-mouse CD3 (145-2C11) BioLegend 100304
Biotin Anti-mouse F4/80 (BM8) BioLegend 123106
Biotin Anti-mouse CD11b (M1/70) BioLegend 101204
Biotin Anti-mouse CD11c (N418) BioLegend 117304
Biotin Anti-mouse c-kit/CD117 (2B8) BioLegend 105804
Biotin Anti-mouse TER-119 (TER-119) BioLegend 116204
Biotin Anti-mouse IgM (II/41) eBioscience 13-5790-85
Biotin Anti-mouse IgD (11-26c) eBioscience 13-5993-85
Biotin Anti-mouse CD93 (AA4.1) eBioscience 13-5892-85
Biotin Anti-mouse Thy1.2/CD90.2 (30-H12) eBioscience 13-0903-85
Biotin Anti-mouse Gr-1/Ly-6G (RB6-8C5) eBioscience 13-5931-85
Biotin Anti-mouse CD5 (53-7.3) eBioscience 13-0051-85
Biotin Anti-mouse CD43 (S7) BD Biosciences 553269
Biotin Anti-mouse CD138 (281-2) BD Biosciences 553713
FITC Anti-mouse IgM (RMM-1) BioLegend 406505
FITC Anti-mouse IgM (II/41) eBioscience 11-5790-85
FITC Anti-mouse IgD (11-26c) BioLegend 405703
FITC Anti-mouse CD5 (53-7.3) BioLegend 100605

	FITC Anti-mouse CD11b (M1/70) BioLegend 101205<br>FITC Anti-mouse CD43 (S11) BioLegend 143203<br>AlexaFluor700 Anti-mouse CD45R/B220 (B220) BioLegend 103232<br>Pacific Blue Anti-mouse CD38 (90) BioLegend 102720<br>Biotin Anti-human IgD (IA6-2) BD Biosciences 555777<br>Biotin Anti-human CD2 (RPA-2.10) BioLegend 300204<br>Biotin Anti-human CD4 (63D3) BioLegend 300504<br>Brilliantviolet786 Anti-human CD19 (H1B19) BioLegend 302240<br>AlexaFluor700 Anti-human CD27 (O323) BioLegend 302814<br>FITC Anti-human IgG (G18-145) BD Biosciences 555786
Validation	All antibodies used in this study are commercially available, and were used with validation procedures by the manufacturers.

Eukaryotic cell lines

Policy information about cell lines

Cell line source(s)	Expi293F cells (Thermofisher, Cat# A14527)<br>NB-21 cells (Kuraoka et al., 2016)<br>MS40L-low cells (Watanabe et al., 2019)<br>MDCK cells (Influenza Virus Research Center, National Institute of Infectious Diseases, Japan)
Authentication	Cell line was not authenticated.
Mycoplasma contamination	Cell lines tested negative for mycoplasma contamination.
Commonly misidentified lines<br>(See ICLAC register)	No commonly misidentified cell lines were used.

Animals and other organisms

Policy information about studies involving animals; ARRIVE guidelines recommended for reporting animal research

Laboratory animals	BALB/c mice were purchased from Japan SLC.<br>NOD/SCID/JAK3-/- mice were kindly provided by Dr. S. Okada (Kumamoto University).<br>All mice were used at a minimum 7 weeks old. Females were used.
Wild animals	This study did not involve wild animals.
Field-collected samples	This study did not involve samples collected from the field.
Ethics oversight	Animal procedures were approved by the Animal Ethics Committee of the National Institute of Infectious Diseases, Japan, and performed in accordance with the guidelines of the Institutional Animal Care and Use Committee.

Note that full information on the approval of the study protocol must also be provided in the manuscript.

Human research participants

Policy information about studies involving human research participants

Population characteristics	Heparinized peripheral blood were obtained from healthy donors.<br>Population characteristics were not available to researchers.
Recruitment	Majority of participants in this study were recruited by Japanese Red Cross Society, and only one participant was recruited by our department as the volunteer.
Ethics oversight	The studies using human samples were approved by the Institutional Ethics Committee of Human Experimentation and performed in accordance with the Ethical Guidelines for Medical and Health Research Involving Human Subjects in Japan.

Note that full information on the approval of the study protocol must also be provided in the manuscript.

Flow Cytometry

Plots

Confirm that:

☒ The axis labels state the marker and fluorochrome used (e.g. CD4-FITC).

☒ The axis scales are clearly visible. Include numbers along axes only for bottom left plot of group (a 'group' is an analysis of identical markers).

☒ All plots are contour plots with outliers or pseudocolor plots.

☒ A numerical value for number of cells or percentage (with statistics) is provided.

Methodology

Sample preparation	【Cell preparation】<br>Lung;<br>Mice were perfused with PBS in the right ventricle to clear the blood from lungs. Lungs were minced and incubated at 37°C for 45-60 min in DMEM (5% FBS, Not containing 2-mercaptoethanol) containing 2 mg/ml collagenase D (Roche) and 10 μg/ml DNase I (Roche) and then disrupted between the frosted ends of glass slides. After centrifugation in a 70%/44%/30% Percoll gradient, the cells at the 70%/44% interface were recovered.<br><br>MLN and Spleen;<br>MLN or spleen were mechanically disrupted between the frosted ends of glass slides. Lysis of RBCs in sample was performed.<br><br>PBMC;<br>PBMC isolation was performed by density centrifugation using Ficoll-Hypaque (GE Healthcare).<br><br>Cells were resuspended in DMEM (2% FBS) and pass through the nylon mesh.<br><br>【Staining】<br>Single cell suspension were plated at ~4x10^6 cells in 96-well u-bottom plates and stained with 100 ul of staining mixtures in DMEM (2% FBS).<br><br>Murine cells were pretreated with anti-FcγRII/III mAb for 10 min on ice, and then incubated with mixtures of biotinylated mAbs for 30 min on ice. Cells were washed with DMEM (2% FBS) .The secondary staining was followed by a mixture of fluorochrome-conjugated antibodies, SA, HA antigen probes, and dead cell stain regents for 30 min on ice. Cells were washed twice with DMEM (2% FBS) and then resuspended in DMEM (2% FBS) or RPMI (10% FBS).<br><br>Human PBMCs were incubated with mixtures of biotinylated mAbs for 30 min on ice and then washed twice with DMEM (2% FBS) ,stained with fluorochrome-conjugated secondary mAbs, HA probes, SA, and dead cell stain regents for 30 min on ice. Cells were washed twice with DMEM (2% FBS) and then resuspended in DMEM (2% FBS) or RPMI (10% FBS).
Instrument	BD FACS Canto II and Aria III (BD)
Software	FACS Diva software (BD), Flowjo v9.9.6 (Tree star Inc.)
Cell population abundance	Single cell sorting was performed in this study.
Gating strategy	All samples were initially gated on the lymphocytes (FSC-A/SSC-A), and then doublets were excluded.<br><br>【Murine sample】<br>Dump- (IgM, IgD, Gr-1, CD3, CD5, CD11b, CD11c, CD43, CD90, CD93, TER-119, F4/80, CD117, and CD138) B220+ live cells were selected. X31 HA+ memory B cells (CD38+) and GC B cells (CD38dull) were gated, and then the strain-specific B cells (X31 HA+ Urg HA-) and the cross-reactive B cells (X31 HA+ Urg HA+) were identified among X31 HA+ memory/GC B cells.<br><br>In some experiments using trimeric HAs as probes;<br>See Supplementary Figure . 4 and Figure. 3b.<br><br>【Human sample】<br>Firstly, Dump- (IgD, CD2, and CD4) CD19+ live cells were selected. After gating on IgG+ memory B cells (CD27+ IgG+), Victoria HA + cells (Victoria HA PE+ Victoria HA AF594+) were gated and then the broadly-reactive B cells (Victoria HA PE+ Victoria HA AF594 + X31 HA+; Figure. 2a) were identified.

☒ Tick this box to confirm that a figure exemplifying the gating strategy is provided in the Supplementary Information.
